# Supplementary material for: Sequence-based prediction of the intrinsic solubility of peptides containing non-natural amino acids
Source: Nat Commun. 2023 Nov 17;14:7475. doi: 10.1038/s41467-023-42940-w (PMC10656490; doi:10.1038/s41467-023-42940-w)
Supplement: Supplementary file 1 — Supplementary Information [file 41467_2023_42940_MOESM1_ESM.pdf]

Supplementary Material

Sequence-based prediction of the solubility of peptides containing non-natural amino acids

Marc Oeller[1], Ryan Kang[1], Hannah Bolt[2], Ana Gomes dos Santos[3], Annika Langborg Weinmann[4], Antonios Nikitidis[5], Pavol Zlatoidsky[5], Wu Su[5], Werngard Czechtizky[5], Leonardo De Maria[5], Pietro Sormanni[1]*, Michele Vendruscolo[1]*

[1]*Centre for Misfolding Diseases, Yusuf Hamied Department of Chemistry, University of Cambridge, Cambridge, UK*
[2]*Discovery Sciences, BioPharmaceuticals R&D, AstraZeneca, Cambridge, UK*
[3]*Pharmaceutical Sciences, BioPharmaceuticals R&D, AstraZeneca, Cambridge, UK*
[4]*Early Chemical Development, Pharmaceutical Sciences, BioPharmaceuticals R&D, AstraZeneca, Gothenburg, Sweden*
[5]*Medicinal Chemistry, Research and Early Development, Respiratory and Immunology, BioPharmaceuticals R&D, AstraZeneca, Gothenburg, Sweden*

*Corresponding Authors: mv245@cam.ac.uk, ps589@cam.ac.uk

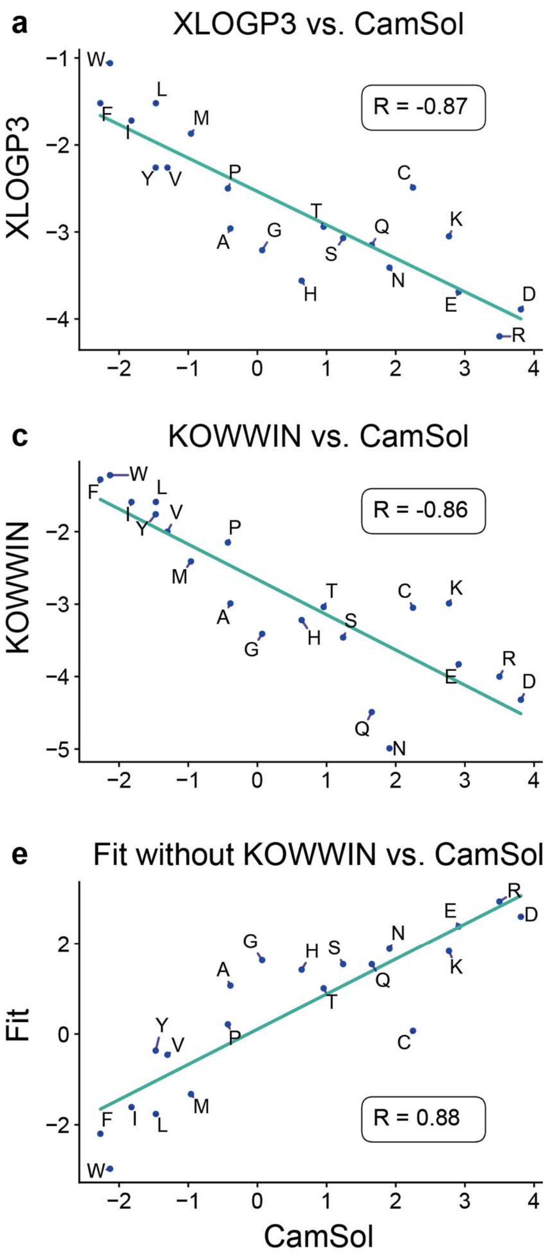

Supplementary Figure 1. Comparison of different hydrophobicity predictors with CamSol hydrophilicity values. Correlation between the hydrophobicity values of XLOGPS **(a)**, ALOGP3 **(b)** and KOWWIN **(c)** and the tabulated hydrophilicity values used in CamSol. **(d)** Values from all three predictors were used to fit a linear regression model to the CamSol hydrophilicity values and then plotted to show the correlation between the fit and the tabulated CamSol hydrophilicity values (Pearson's coefficient of correlation = 0.9). **(e)** Fit without KOWWIN against CamSol hydrophilicity values (Pearson's coefficient of correlation = 0.88).

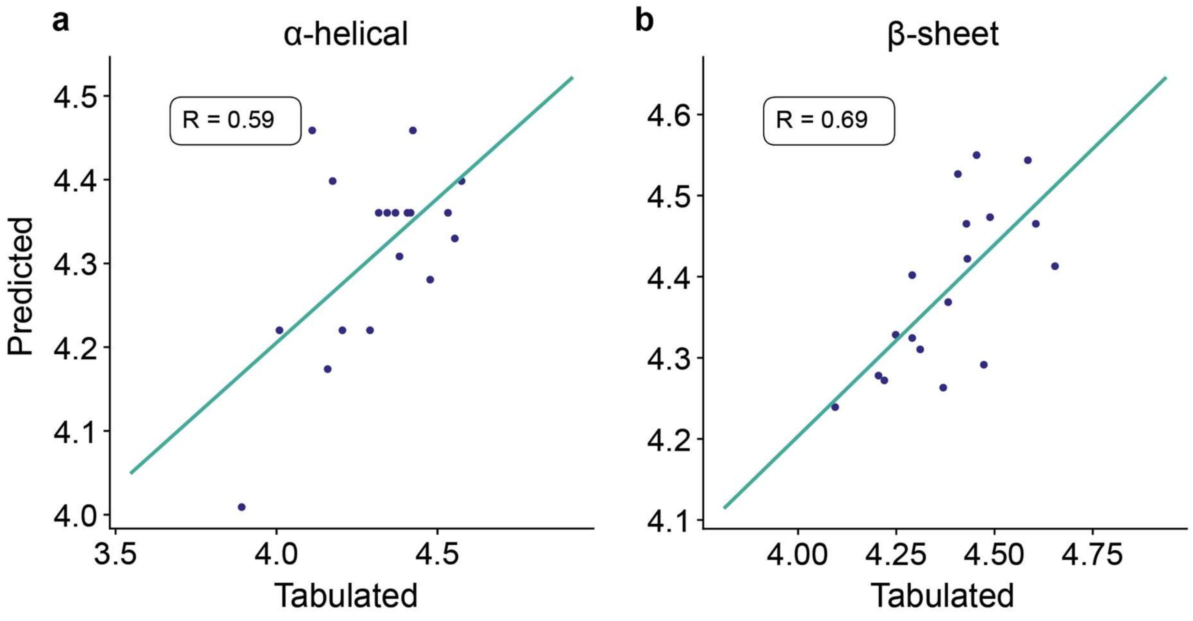

Supplementary Figure 2. Correlation between predicted and tabulated secondary structure propensities. The secondary structure propensity predictor predicts the α-helical propensity **(a)** and β-sheet propensity **(b)** for the 20 natural amino acids with Pearson's coefficients of correlation of 0.59 and 0.69, respectively.

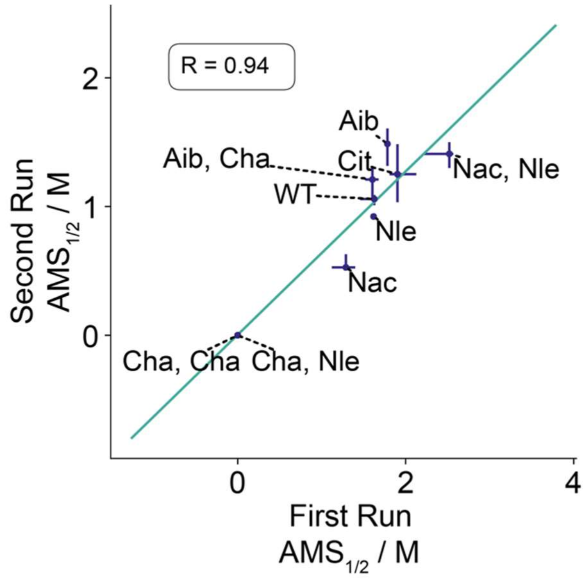

Supplementary Figure 3. Comparison of independent AMS runs. To verify that replacing PEG with AMS continues to yield reliable and replicable results we performed two independent solubility experiments with AMS on two separate days. The correlation indicates that replacing PEG with AMS is feasible.

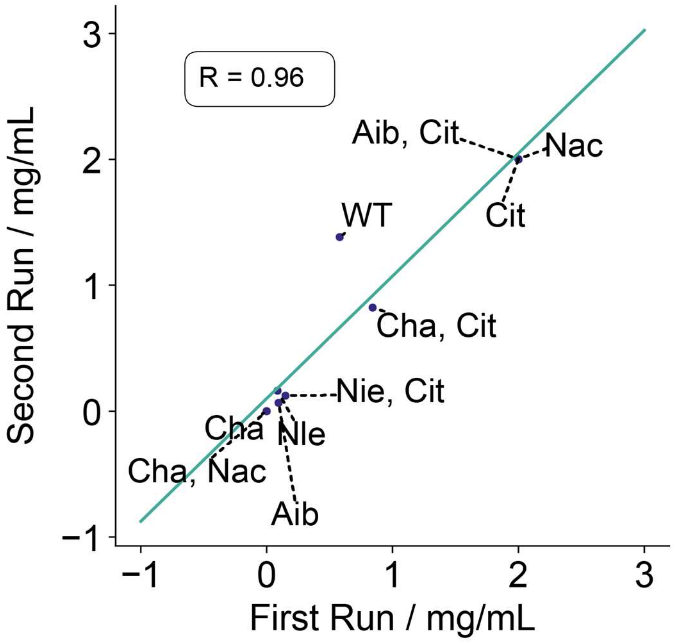

Supplementary Figure 4. Comparison of independent ultracentrifugation runs. To verify the reproducibility of the ultracentrifugation method we performed two independent experiments on different days. The high correlation shows that the results from the ultracentrifugation assay are reliable.

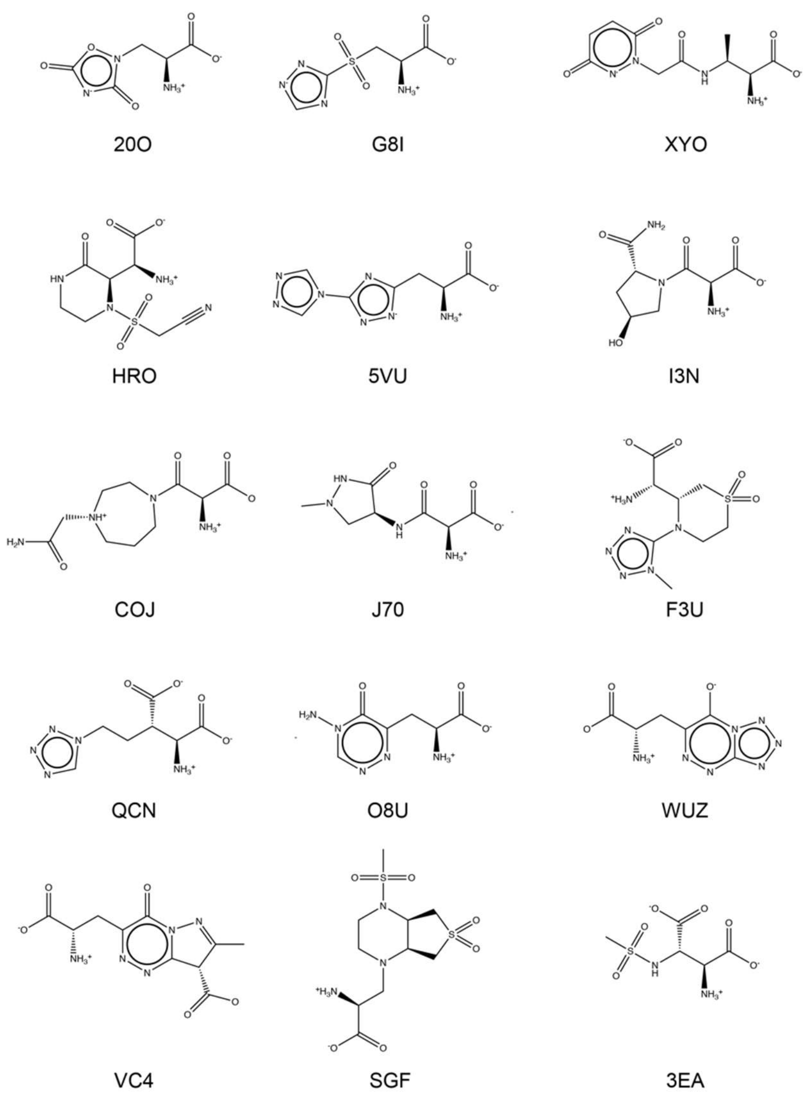

Supplementary Figure 5. Selection of mAAs that most effectively promote solubility. By analysing the positive tail end of the solubility prediction distribution, we found that most mAAs that have a positive effect on solubility contain many hydrogen bonding atoms such as nitrogen and oxygen.

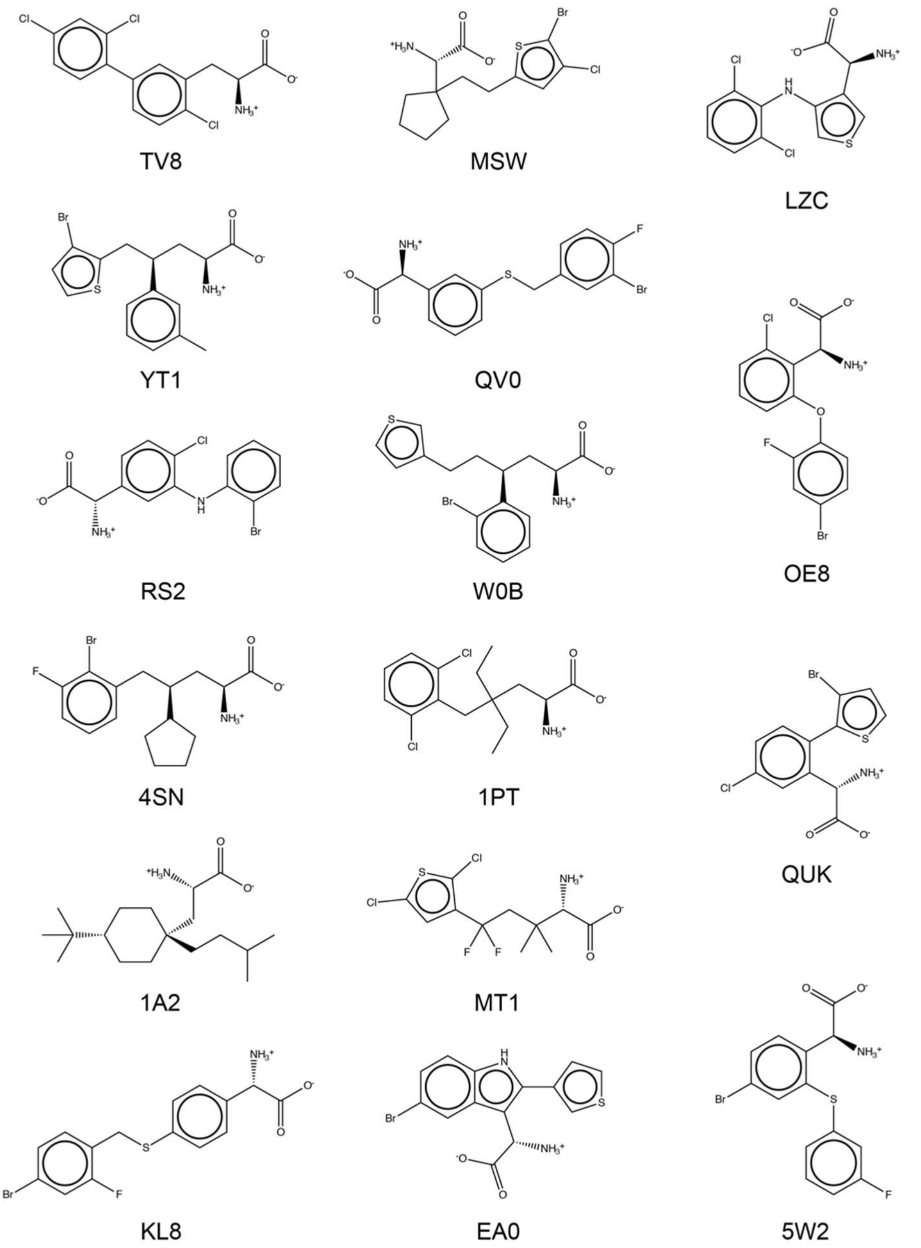

Supplementary Figure 6. Selection of mAAs that most effectively decrease solubility. By analysing the negative tail end of the solubility prediction distribution, we found that most mAAs that have a negative effect on solubility contain several aromatic rings and often halogens such as chlorine or bromine.
