# Supplementary material for: Sequence-based prediction of the intrinsic solubility of peptides containing non-natural amino acids
Source: Nat Commun. 2023 Nov 17;14:7475. doi: 10.1038/s41467-023-42940-w (PMC10656490; doi:10.1038/s41467-023-42940-w)
Supplement: Supplementary file 3 — Description of Additional Supplementary Files [file 41467_2023_42940_MOESM3_ESM.pdf]

Description of Additional Supplementary Files

File Name: Supplementary data 1.
Description: Included as a separate MS Excel file. Contains detailed information on the preparation and purification of the peptides.
