# Supplementary material for: Sequence-based prediction of the intrinsic solubility of peptides containing non-natural amino acids
Source: Nat Commun. 2023 Nov 17;14:7475. doi: 10.1038/s41467-023-42940-w (PMC10656490; doi:10.1038/s41467-023-42940-w)
Supplement: Supplementary file 5 — Reporting Summary [file 41467_2023_42940_MOESM5_ESM.pdf]

nature portfolio

Corresponding author(s): Michele Vendruscolo

Last updated by author(s): Sep 13, 2023

Reporting Summary

Nature Portfolio wishes to improve the reproducibility of the work that we publish. This form provides structure for consistency and transparency in reporting. For further information on Nature Portfolio policies, see our Editorial Policies and the Editorial Policy Checklist.

Statistics

For all statistical analyses, confirm that the following items are present in the figure legend, table legend, main text, or Methods section.

n/a	Confirmed	
☐	☒	The exact sample size (*n*) for each experimental group/condition, given as a discrete number and unit of measurement
☐	☒	A statement on whether measurements were taken from distinct samples or whether the same sample was measured repeatedly
☒	☐	The statistical test(s) used AND whether they are one- or two-sided<br>*Only common tests should be described solely by name; describe more complex techniques in the Methods section.*
☒	☐	A description of all covariates tested
☒	☐	A description of any assumptions or corrections, such as tests of normality and adjustment for multiple comparisons
☐	☒	A full description of the statistical parameters including central tendency (e.g. means) or other basic estimates (e.g. regression coefficient) AND variation (e.g. standard deviation) or associated estimates of uncertainty (e.g. confidence intervals)
☒	☐	For null hypothesis testing, the test statistic (e.g. *F*, *t*, *r*) with confidence intervals, effect sizes, degrees of freedom and *P* value noted<br>*Give P values as exact values whenever suitable.*
☒	☐	For Bayesian analysis, information on the choice of priors and Markov chain Monte Carlo settings
☒	☐	For hierarchical and complex designs, identification of the appropriate level for tests and full reporting of outcomes
☐	☒	Estimates of effect sizes (e.g. Cohen's *d*, Pearson's *r*), indicating how they were calculated

Our web collection on statistics for biologists contains articles on many of the points above.

Software and code

Policy information about availability of computer code

Data collection	Python 3.6, CamSol 3.0, CamSolPTM, pIChemiSt, ALOGPS 2.1, XLOGP3, KOWWIN; The software is free of charge for academic users under https://www-cohsoftware.ch.cam.ac.uk/index.php/camsolptm. All experimental data will be made available upon request.
Data analysis	Python 3.6

For manuscripts utilizing custom algorithms or software that are central to the research but not yet described in published literature, software must be made available to editors and reviewers. We strongly encourage code deposition in a community repository (e.g. GitHub). See the Nature Portfolio guidelines for submitting code & software for further information.

Data

Policy information about availability of data

All manuscripts must include a data availability statement. This statement should provide the following information, where applicable:

- Accession codes, unique identifiers, or web links for publicly available datasets
- A description of any restrictions on data availability
- For clinical datasets or third party data, please ensure that the statement adheres to our policy

All peptide sequences are given in Table 2 and Supplementary Data 1. All data necessary to replicate, evaluate or extend the research presented in this article are provided throughout the article, the supporting information and the Source Data file. All predicted values are provided in the Source Data file and can be replicated

by using the webserver under https://www-cohsoftware.ch.cam.ac.uk/index.php/camsolptm. Information on peptide production and purification are included in the supporting information.

Human research participants

Policy information about studies involving human research participants and Sex and Gender in Research.

Reporting on sex and gender	N/A
Population characteristics	N/A
Recruitment	N/A
Ethics oversight	N/A

Note that full information on the approval of the study protocol must also be provided in the manuscript.

Field-specific reporting

Please select the one below that is the best fit for your research. If you are not sure, read the appropriate sections before making your selection.

☒ Life sciences ☐ Behavioural & social sciences ☐ Ecological, evolutionary & environmental sciences

For a reference copy of the document with all sections, see nature.com/documents/nr-reporting-summary-flat.pdf

Life sciences study design

All studies must disclose on these points even when the disclosure is negative.

Sample size	We screened a large number (>50,000) variants in silico. From those we produced and verified 37 peptides. The number was chosen as a compromise between production/purification capacity and number of variants. More details on how and why we chose these variants can be found under Results: Peptide design
Data exclusions	No data was excluded.
Replication	The main experimental method was extensively validated previously (Oeller et al Sci Reports 2021). For the AMS protocol and the ultracentrifugation experiments, we verified the measurements by running 2 independent experiments (different batches, buffer, and performed days apart).
Randomization	Randomization was not applicable for this study as none of the experimental methods demonstrate any bias for the order in which samples are measured.
Blinding	Blinding was not applicable for this study as predictions needed to compared to experimental results and therefore clear labeling of samples is necessary.

Reporting for specific materials, systems and methods

We require information from authors about some types of materials, experimental systems and methods used in many studies. Here, indicate whether each material, system or method listed is relevant to your study. If you are not sure if a list item applies to your research, read the appropriate section before selecting a response.

Materials & experimental systems

n/a	Involved in the study	
☒	☐	Antibodies
☒	☐	Eukaryotic cell lines
☒	☐	Palaeontology and archaeology
☒	☐	Animals and other organisms
☒	☐	Clinical data
☒	☐	Dual use research of concern

Methods

n/a	Involved in the study	
☒	☐	ChIP-seq
☒	☐	Flow cytometry
☒	☐	MRI-based neuroimaging
